# Dietary advanced glycation end products intake and osteoporosis risk in adults with type 2 diabetes mellitus

**DOI:** 10.1097/MD.0000000000050261

**Published:** 2026-09-11

**Authors:** Liping Jin, Qing Liu, Shuai Qin, Lu Gan, Xiaoqiong Chen, Mei Mo, Jialu Liao, Yuanyuan Chen

**Affiliations:** a Endocrinology Department, The First Affiliated Hospital of Guizhou University of Chinese Medicine, Guiyang, Guizhou Province, China; b Oncology Department, The First Affiliated Hospital of Guizhou University of Chinese Medicine, Guiyang, Guizhou Province, China.

**Keywords:** Advanced glycation end products, bone turnover markers, dietary intake, inflammation, osteoporosis, type 2 diabetes mellitus

## Abstract

Advanced glycation end products (AGEs) derived from dietary sources may aggravate metabolic inflammation and impair bone turnover in patients with type 2 diabetes mellitus (T2DM); however, clinical evidence linking dietary AGEs exposure to osteoporosis (OS) in T2DM remains limited. In this retrospective cohort study, 200 adults with T2DM treated at a tertiary hospital between January 2020 and December 2022 were included. Dietary AGEs intake at baseline was quantified using a simplified food frequency questionnaire complemented by a 3-day dietary record and categorized as high (≥75th percentile), moderate (50th–<75th percentile), or low (<50th percentile). Osteoporosis and osteopenia were defined by dual-energy X-ray absorptiometry T-scores. Inflammatory markers (procalcitonin [PCT], interleukin-6 [IL-6], and C-reactive protein [CRP]) were assessed at baseline and at each patient’s last available follow-up. Logistic regression was performed to identify factors independently associated with OS, and receiver operating characteristic (ROC) curves evaluated predictive performance. The incidence of OS differed across dietary AGEs categories (45.0% high, 23.9% moderate, 18.6% low; *P* = .013). At the last available follow-up, the low-intake group exhibited lower PCT, IL-6, and CRP levels than the high- and moderate-intake groups (all *P* < .05). Multivariable analysis identified PCT, β-CTX, P1NP, and dietary AGEs intake as independent factors associated with OS (all *P* < .05). Dietary AGEs intake showed acceptable discrimination for OS (AUC = 0.797). Higher dietary AGEs intake was associated with a greater likelihood of osteoporosis in adults with T2DM and may contribute through inflammation-related and bone turnover-related pathways.

## 1. Introduction

Muscles and bones, as crucial components of the musculoskeletal system, both originate from the mesoderm, share common mesenchymal precursors, and are adjacent in anatomical location. They exhibit similarities in genetic regulation, paracrine and endocrine mechanisms, and molecular signaling pathways.^[1]^ This close association necessitates the exploration of the relationship between the two and intervention in conditions of muscle loss and bone loss, which is of significant importance in reducing disability and mortality rates among the elderly and enhancing quality of life. Osteoporosis (OS) and muscle loss are common chronic musculoskeletal diseases in the elderly. With the accelerating global aging population, the incidence of both conditions is increasing annually, leading to significant risks of falls and fractures and increased hospitalization rates. Muscle loss manifests as a decrease in muscle mass, strength, or physiological function with aging.^[2]^ OS is a systemic metabolic bone disease characterized by reduced bone mass and microstructural damage, leading to bone fragility and increased susceptibility to fractures.^[3]^ Epidemiological studies show that diabetes mellitus can induce diabetic osteoporosis, exacerbating the burden of fractures. Both type 1 and type 2 diabetes mellitus (T2DM) and certain antidiabetic medications are associated with an increased risk of fractures.^[4]^ Skeletal muscle plays a crucial role in energy metabolism and glucose uptake, and the prevalence of muscle loss in diabetic patients ranges from 7% to 29.3%.^[5]^ Currently, screening for OS in diabetic patients is widely conducted, but diabetic-related muscle loss has not received widespread attention, and the relationship between the two is not well understood, laying the foundation for further exploration in previous studies. T2DM and OS are common metabolic diseases in the elderly. OS is influenced by factors such as age and hormones, leading to reduced bone mass, structural damage, increased bone fragility, and a substantial economic burden to the nation due to fragility fractures.^[6]^ Although many risk factors for OS have been identified and treatment drugs developed, the actual proportion of treated patients is small.^[7]^ A cross-sectional study in China showed that there are over 74.22 million T2DM patients, and sustained hyperglycemia is positively correlated with the risk of OS. Patients with a long disease course are at higher risk of falls and fractures due to various factors,^[8]^ making the management of both conditions a matter of great concern. Poor nutritional status is an important risk factor for the occurrence and development of T2DM and OS. Advanced glycation end products (AGEs) in the diet are harmful substances produced during food processing, and their accumulation is associated with decreased bone mass. High blood sugar in T2DM patients leads to a more significant generation and accumulation of AGEs, which may exacerbate bone metabolism disorders, increase the risk of OS, and indirectly impact skeletal health by influencing muscle metabolism. Currently, there is insufficient research on the relationship between dietary AGEs intake and the occurrence of OS in T2DM patients, with mechanisms awaiting clarification. This study aims to explore the association between the two and provide a basis and strategies for prevention and treatment.

### 1.1. Research objects

This study was approved by the Ethics Committee of The First Affiliated Hospital of Guizhou University of Chinese Medicine. This retrospective cohort study consecutively enrolled adults with type 2 diabetes mellitus (T2DM) who received care at a tertiary hospital between January 2020 and December 2022. Eligible participants were required to meet established diagnostic criteria for T2DM, be aged 18 to 59 years, and have complete baseline clinical and laboratory data available for analysis, including dietary assessment records and bone mineral density (BMD) measurements. All included patients received standardized glucose-lowering management during routine clinical practice according to institutional protocols. Patients were excluded if they had cognitive impairment or communication barriers precluding reliable dietary assessment, had conditions or comorbidities expected to substantially influence systemic inflammation or bone metabolism (e.g., autoimmune disorders or major cardiovascular disease), were pregnant or lactating, had incomplete or non-verifiable key variables (including exposure, outcome, or essential covariates), or lacked sufficient follow-up information to ascertain osteoporosis status. Osteoporosis (OS) and osteopenia were defined using dual-energy X-ray absorptiometry-derived T-scores at the lumbar spine and/or hip. Follow-up was based on routinely scheduled outpatient visits and electronic medical records rather than a prespecified research visit schedule. The observation period began at baseline admission and ended at the last available follow-up record within the study period. Consequently, follow-up duration and visit intervals varied among participants. Patients without adequate follow-up information to determine osteoporosis status were excluded before the formation of the final analytic cohort; therefore, no additional loss to follow-up occurred within the 200 patients included in the analysis. All data were collected and handled in accordance with institutional ethical requirements and the Declaration of Helsinki.

### 1.2. Research methods

#### 1.2.1. Collection of general admission data

At hospital admission, baseline demographic and clinical characteristics were abstracted from the electronic medical record, including age, sex, anthropometric indices (body mass index, BMI), vital signs (blood pressure), and glycemic-related parameters (e.g., blood glucose). These variables were recorded as part of routine clinical assessment and were used to characterize the study population and as candidate covariates in subsequent analyses.

#### 1.2.2. Collection of patients’ laboratory indicators

On the second morning after admission (T0), fasting venous blood samples were collected to measure procalcitonin (PCT), interleukin-6 (IL-6), C-reactive protein (CRP), and bone turnover markers (β-CTX and P1NP). These indicators were reassessed at each patient’s last available follow-up visit (T1). Because this was a retrospective study based on routine care, the interval between T0 and T1 was not standardized.

#### 1.2.3. Measurement of dietary AGEs intake

A simplified Food Frequency Questionnaire (FFQ)^[9]^ was used at admission to obtain information on habitual food intake during the preceding 6 months. The FFQ was complemented by a 3-day dietary record to validate intake frequency and quantify the daily consumption of major food categories. Daily dietary AGEs intake was estimated as follows: Σ (daily intake of a food item × the median AGE concentration assigned to that prepared food item).^[10]^ The AGE values were obtained from the published food AGE database, in which values correspond to foods as consumed or prepared. Cooking method was therefore considered indirectly when a prepared-food entry matching the reported item was available. However, the questionnaire and retrospective records did not consistently capture detailed preparation variables such as exact cooking temperature, duration, browning level, or repeated reheating; these factors were not modeled separately. Participants were categorized according to the distribution of estimated intake: high (≥75th percentile), moderate (50th to < 75th percentile), and low (<50th percentile).

#### 1.2.4. Criteria for diagnosing OS^[11]^

Osteoporosis was diagnosed based on bone mineral density (BMD) equal to or below −2.5 standard deviations (SD) from the mean of young healthy individuals (T-score), or when the T-score of lumbar spine or hip BMD falls between −1.0 and −2.5 SD: diagnosed as osteopenia (low bone mass).

### 1.3. Observational indicators

The observational indicators were: the occurrence of OS and osteopenia in the high-, moderate-, and low-dietary-AGE groups; and changes in inflammatory markers from admission (T0) to the last available follow-up (T1) across the three dietary AGE intake groups.

### 1.4. Statistical analysis

Data were analyzed using SPSS version 27.0 (IBM Corporation, Armonk, NY, USA). Normality was assessed using the Shapiro–Wilk test. Normally distributed continuous variables are presented as mean ± standard deviation. Between-group comparisons were performed using independent-samples *t*-tests for two groups and one-way analysis of variance for three groups. Categorical variables are presented as frequencies and percentages and were compared using the χ^2^ test or Fisher’s exact test, as appropriate. Factors associated with OS in patients with T2DM were examined using univariate analyses followed by binary Logistic regression. Receiver operating characteristic (ROC) curves were used to evaluate the predictive performance of dietary AGEs intake and the combined model. A two-sided *P* value < .05 was considered statistically significant.

## 2. Results

### 2.1. Comparison of OS and reduced bone mass incidence rates among patients with high, moderate, and low dietary AGEs intake

There were 40 cases in the high intake group, and 18 cases developed OS, with an incidence rate of 45.00%; 117 cases in the medium intake group, and 28 cases developed OS, with an incidence rate of 23.93%; and 43 cases in the low intake group, and 8 cases developed OS, with an incidence rate of 18.60%. It can be seen from the data that as the intake of dietary AGEs increases, the incidence of OS in patients shows an increasing trend. There were statistically significant differences in the incidence of OS in patients with AGEs intake in different diets (*P* < .05). In terms of the incidence of bone loss, 19 patients in the high intake group had bone loss, accounting for 47.50%; 33 patients in the medium intake group, accounting for 28.21%; and 12 patients in the low intake group, accounting for 27.91%. Although the *P* value for comparing the incidence of bone loss among different dietary AGEs intake groups was 0.063, there was no significant difference (*P* > .05), as shown in Table 1.

**Table 1:** Comparison of OS and incidence of osteopenia (%).

	Case count	OS	Reduced bone mass
High intake group	40	18 (45.00)	19 (47.50)
Moderate intake group	117	28 (23.93)	33 (28.21)
Low intake group	43	8 (18.60)	12 (27.91)
c^2^ value		8.672	5.522
*P* value		.013	.063

### 2.2. Changes in inflammatory factors among three groups of patients at admission and at the end of follow-up

Comparison of inflammatory factors among the three groups at T0 showed no statistically significant differences (*P* > .05). However, at the end of the follow-up, patients in the low intake group exhibited lower levels of inflammatory factors compared to the high intake and moderate intake groups, with statistically significant inter-group differences (*P* < .05), as shown in Table 2.

**Table 2:** Changes in inflammatory factors.

Indicator		High intake group (n = 40)	Moderate intake group (n = 117)	Low intake group (n = 43)	*F* value	*P* value
PCT (ng/mL)	T0	0.43 ± 0.11	0.41 ± 0.08	0.42 ± 0.07	0.883	.415
	T1	0.23 ± 0.06	0.20 ± 0.07	0.16 ± 0.04	13.131	<.001
IL-6 (pg/mL)	T0	2.84 ± 0.95	2.83 ± 0.82	2.82 ± 0.61	0.006	.994
	T1	1.42 ± 0.34	1.32 ± 0.37	1.16 ± 0.29	6.009	.003
CRP (mg/mL)	T0	12.03 ± 1.17	11.92 ± 1.19	12.14 ± 1.08	0.593	.554
	T1	9.19 ± 0.98	8.82 ± 0.77	7.54 ± 1.02	5.787	.004

CRP = C-reactive protein, IL-6 = Interleukin-6, PCT = Procalcitonin.

### 2.3. Univariate analysis of factors influencing OS occurrence among subgroups of T2DM patients

There was no significant difference between the two groups in terms of age, gender, BMI, hypertension, smoking, alcohol consumption, type of medication, duration of diabetes, TP, TG, ALB, Cr, TC, HDL-C, LDL-C, or CRP (*P >* .05).Patients were divided into OS and non-OS groups based on the OS occurrence during follow-up. A comparison of PCT, IL-6, β-CTX, P1NP, and dietary AGEs intake between the two groups showed statistically significant differences (*P *< .05), as shown in Table 3.

**Table 3:** Univariate analysis of factors influencing OS occurrence in T2DM patients.

Indicator		OS Group (n = 54)	Non-OS Group (n = 146)	*t/χ*^2^ value	*P* value
Age (years)		57.54 ± 10.24	58.19 ± 11.25	0.371	.711
Gender	Male	29	77	0.015	.904
	female	25	69		
BMI (kg/m^2^)		22.54 ± 1.36	22.49 ± 1.28	0.241	.810
Hypertension	Yes	13	32	0.105	.746
	No	41	114		
Smoking	Yes	18	42	0.391	.532
	No	36	104		
Alcohol Intake	Yes	22	52	0.444	.505
	No	32	94		
Medication Type	Single	17	50	0.135	.713
	Combination	37	96		
Duration of T2DM (years)		6.82 ± 1.25	6.94 ± 1.37	0.563	.574
TP (g/L)		71.79 ± 10.54	71.25 ± 11.08	0.310	.757
TG (mmol/L)		1.55 ± 0.12	1.52 ± 0.11	1.670	.096
ALB (g/L)		41.75 ± 6.61	40.73 ± 5.84	1.058	.292
Cr (μmol/L)		64.85 ± 10.11	65.28 ± 10.41	0.261	.794
TC (mmol/L)		4.43 ± 0.72	4.58 ± 0.71	1.321	.188
HDL-C (mmol/L)		1.26 ± 0.37	1.23 ± 0.21	0.717	.474
LDL-C (mmol/L)		2.57 ± 1.28	2.40 ± 1.54	0.724	.470
PCT (ng/mL)		0.45 ± 0.11	0.31 ± 0.07	10.638	<.001
IL-6 (pg/mL)		2.96 ± 0.92	2.69 ± 0.81	2.016	.045
CRP (mg/mL)		12.03 ± 1.21	11.90 ± 1.37	0.614	.540
β-CTX (μg/L)		56.35 ± 7.85	45.87 ± 7.92	8.328	<.001
P1NP (μg/L)		0.75 ± 0.11	0.59 ± 0.07	12.158	<.001
Dietary AGEs Intake	High	18	22	8.672	.013
	Medium	28	89		
	Low	8	35		

All analysis indicators represent data at admission.

ALB = albumin, BMI = body mass index, Cr = creatinine, CRP = C-reactive protein, HDL-C = high-density lipoprotein cholesterol, IL-6 = interleukin-6, LDL-C = low-density lipoprotein cholesterol, PCT = procalcitonin, P1NP = procollagen I N-terminal propeptide, TC = total cholesterol, TG = triglyceride, TP = total protein, β-CTX = β-CrossLaps (serum).

### 2.4. Binary logistics regression analysis of factors influencing OS occurrence in T2DM patients

Variables that were statistically significant in the univariate analysis were entered as independent variables in a binary Logistic regression model, with OS occurrence as the dependent variable (OS = 1, non-OS = 0). The analysis indicated that PCT, β-CTX, P1NP, and dietary AGEs intake were independently associated with OS in patients with T2DM (all *P* < .05), as shown in Tables 4 and 5.

**Table 4:** Variable assignment.

Influencing factor	Assignment
PCT	Original value
IL-6	Original value
β-CTX	Original value
P1NP	Original value
Dietary AGEs intake	Low = 0, Medium = 1, High = 2

**Table 5:** Binary logistic regression analysis influencing factors.

Factor	β	Standard Error	Wald	*P*	Exp(β)	95%CI
PCT	0.497	0.237	4.389	<.001	1.643	1.033~2.614
IL-6	0.201	0.182	1.223	.241	1.223	0.856~1.747
β-CTX	0.852	0.382	4.978	<.001	2.345	1.109~4.958
P1NP	0.782	0.379	4.253	<.001	2.185	1.040~4.593
Dietary AGEs Intake	0.248	0.125	3.950	<.001	1.282	1.003~1.638

### 2.5. ROC curve analysis of indicators for predicting OS in T2DM patients

**Figure 1.**
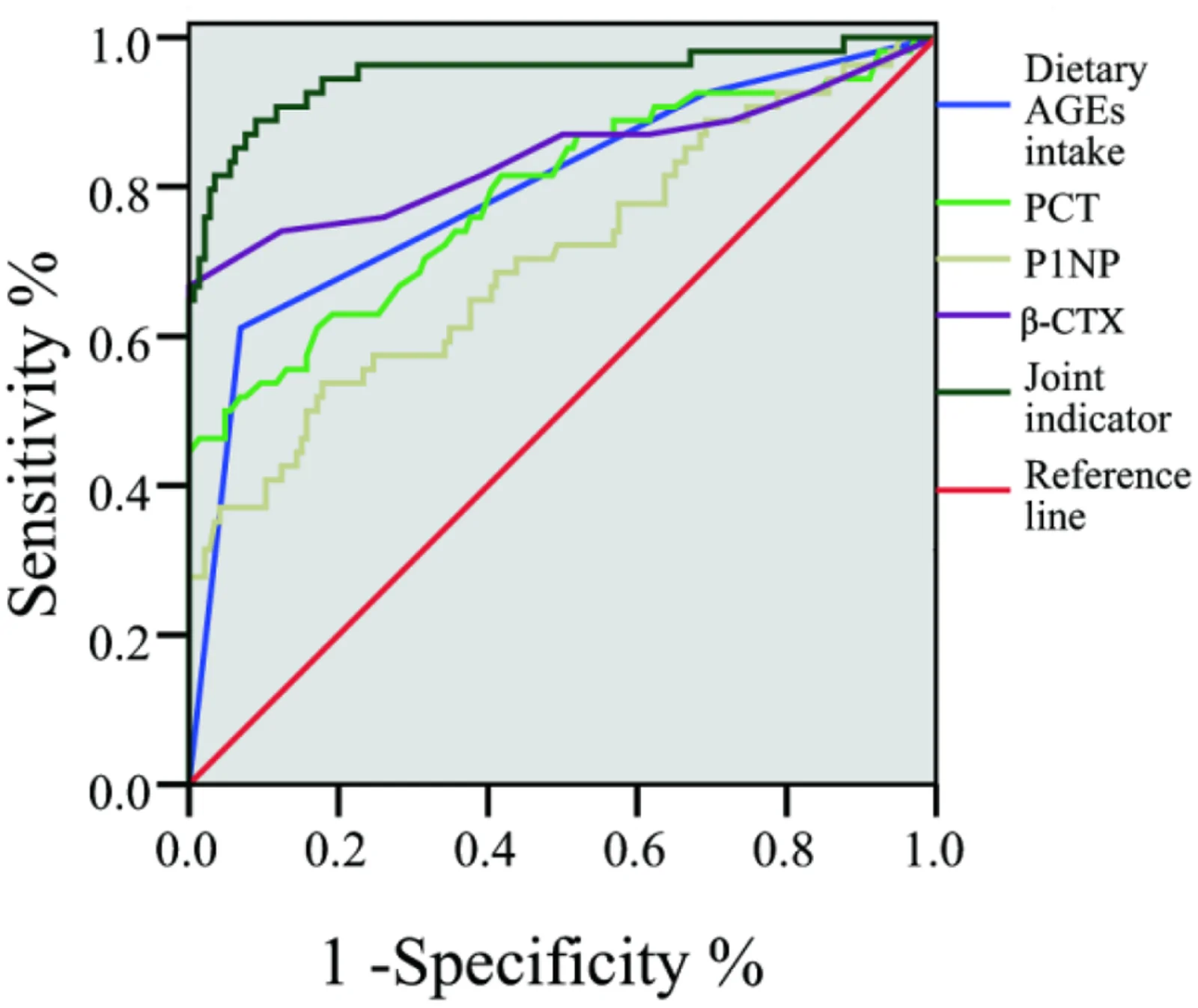
ROC curve.

The ROC analysis results indicated that the predicted AUC for dietary AGEs intake was 0.797, with a standard error of 0.039 (95% CI: 0.721–0.873) and a Youden index of 0.54, yielding a sensitivity of 61.11% and specificity of 93.15%. When combined with other indicators, the AUC of the predictive curve increases to 0.950, with a standard error of 0.021 (95% CI: 0.909–0.992) and a Youden index of 0.80, resulting in a sensitivity of 88.89% and specificity of 91.11%. See Table 6 and Figure 1 for details.

**Table 6:** ROC curve analysis results.

Indicator	AUC	SE	95%CI	Youden	Sensitivity	Specificity
PCT	0.788	0.040	0.709~0.867	0.45	50.00	95.21
β-CTX	0.840	0.040	0.761~0.919	0.62	74.11	87.78
P1NP	0.705	0.045	0.617~0.792	0.36	53.70	82.19
Dietary AGEs Intake	0.797	0.039	0.721~0.873	0.54	61.11	93.15
Combined Indicators	0.950	0.021	0.909~0.992	0.80	88.89	91.11

## 3. Discussion

This study found that higher dietary AGEs intake was associated with a greater occurrence of osteoporosis in adults with T2DM. The association remained significant after adjustment for other variables, and the findings were accompanied by differences in inflammatory and bone turnover markers.

At the last available follow-up, the high-intake group had higher PCT, IL-6, and CRP levels than the low-intake group. These findings are consistent with the hypothesis that dietary AGEs may contribute to chronic low-grade inflammation, which can favor osteoclast activation, inhibit osteoblast function, and disturb bone remodeling.^[12]^ Dietary AGEs may also enter the circulation after intestinal absorption and contribute to AGE accumulation in the bone matrix, potentially impairing collagen quality and bone mechanical properties. This interpretation is supported by broader evidence linking nutrition and the gut-bone axis to skeletal health^[13–15]^; however, the observational design of the present study does not establish causality.

Further binary logistic regression analysis revealed that PCT, β-CTX, P1NP, and dietary AGEs intake are independent influencing factors for OS occurrence in T2DM patients. The interference of AGEs intake on the balance of bone turnover is further confirmed. AGEs activate the receptor for advanced glycation end products (RAGE) signaling pathway, leading to a bidirectional imbalance in the bone turnover process. AGEs inhibit bone formation by upregulating the expression of sclerostin (SOST) to inhibit the Wnt/β-catenin pathway, reducing the expression of osteogenic differentiation markers (such as RUNX2 and ALP), and inducing apoptosis of osteoblasts, thus decreasing the rate of bone formation. On the other hand, although the role of AGEs in bone resorption is controversial, long-term accumulation can promote bone resorption by activating the JNK and NF-κB pathways.^[16,17]^ This intricate regulatory mechanism results in overall bone turnover suppression, with an unclear direction, impeding the normal renewal and repair processes of bones, consequently leading to osteoporosis. Abnormal changes in β-CTX and P1NP levels may be external manifestations of AGEs disrupting the balance of bone turnover, further emphasizing the role of AGEs intake in the pathogenesis of OS in T2DM. Previous studies, such as that by Quattrini S et al,^[18]^ have also demonstrated the preventive effect of the Mediterranean diet (low in AGEs) on osteoporosis, aligning closely with the results of this study, further substantiating the findings.

Dietary AGEs intake alone showed acceptable discrimination for OS (AUC = 0.797), although its sensitivity was modest (61.11%). The combined model achieved a higher AUC (0.950), suggesting that dietary exposure should be interpreted together with inflammatory and bone turnover markers rather than as a stand-alone predictor. Because the model was developed and evaluated in the same retrospective sample, its performance may be optimistic and requires external validation.

These findings support further evaluation of dietary AGE reduction as a potentially modifiable component of osteoporosis prevention in T2DM. Practical approaches may include favoring moist, lower-temperature cooking methods and limiting heavily browned, grilled, fried, or repeatedly reheated foods, while maintaining adequate protein and overall nutritional quality. Nevertheless, the present study has several limitations. Follow-up intervals and duration were not standardized because data were derived from routine care, and patients without adequate outcome ascertainment were excluded. In addition, detailed cooking temperature, duration, browning, and reheating information was not consistently available; cooking method was represented only indirectly through prepared-food values in the AGE database. Dietary intake was assessed at baseline and may have changed over time, residual confounding cannot be excluded, and the single-center retrospective design limits generalizability. Prospective multicenter studies with standardized follow-up, repeated dietary assessment, and explicit cooking-method quantification are needed.

## 4. Conclusion

Higher dietary AGEs intake was associated with a greater likelihood of osteoporosis in adults with T2DM and was accompanied by differences in inflammatory and bone turnover markers. As a potentially modifiable exposure, dietary AGEs may be relevant to risk assessment and prevention; however, the retrospective design precludes causal inference. Future research should prospectively quantify cooking methods, standardize follow-up, validate the predictive model externally, and examine whether reducing dietary AGEs improves bone-related outcomes.

## Author contributions

**Conceptualization:** Liping Jin, Qing Liu, Shuai Qin, Lu Gan, Xiaoqiong Chen, Mei Mo, Jialu Liao, Yuanyuan Chen.

**Data curation:** Liping Jin, Qing Liu, Shuai Qin, Lu Gan, Xiaoqiong Chen, Mei Mo, Jialu Liao, Yuanyuan Chen.

**Formal analysis:** Liping Jin, Qing Liu, Shuai Qin, Lu Gan, Xiaoqiong Chen, Mei Mo, Jialu Liao, Yuanyuan Chen.

**Funding acquisition:** Liping Jin, Yuanyuan Chen.

**Investigation:** Liping Jin.

**Writing – original draft:** Liping Jin.

**Writing – review & editing:** Liping Jin.
